# Chimeric antigen receptor T-cell immunotherapy in breast cancer: development and challenges

**DOI:** 10.7150/jca.54095

**Published:** 2021-01-01

**Authors:** Sara Toulouie, Gary Johanning, Yihui Shi

**Affiliations:** 1 California Northstate University, College of Medicine, Elk Grove CA, USA.; 2 SunnyBay Biotech, Fremont, CA USA.

**Keywords:** breast cancer, T-cell, immunotherapy, chimeric antigen receptor, TNBC

## Abstract

Chimeric antigen receptor (CAR) T-cell therapy is an innovative form of immunotherapy wherein autologous T-cells are genetically modified to express chimeric receptors encoding an antigen-specific single-chain variable fragment and costimulatory molecules. Moreover, CAR T-cell therapy can only work successfully in patients who have an intact immune system. Therefore, patients receiving cytotoxic chemotherapy will be immunosuppressed making CAR-T therapy less effective. In adoptive CD8+ T-cell therapy (ACT), numerous tumor-specific, engineered T-cells are sourced from patients, expanded *in vitro*, and infused back expressing tumor-specific antigen receptors. The most successful ACT, anti-CD19 chimeric antigen receptor T-cell therapy directed against B-cell lymphoma, has proved to be efficacious. However, current efforts to utilize this approach for solid tumors, like breast cancer, have shown only modest improvement. Nevertheless, the potential efficacy of CAR-T therapy is promising in an era of immunological advances. By appropriately manipulating CAR T-cells to combat the immunosuppressive forces of the tumor microenvironment, significant eradication of the solid tumor may occur. This review discusses CAR T-cell therapy and its specificity and safety in adoptive cell transfers in breast cancer. We will highlight novel discoveries in CAR T-cell immunotherapy and the formidable barriers including suppression of T-cell function and localization at tumor sites.

## Introduction

Breast cancer has consistently been characterized as a leading cause of death in women. One-fourth of all women with cancer, or 1.5 million women, are diagnosed with breast cancer each year throughout the world 1.This illness is often diagnosed during a routine screening or incidentally and may spread to lymph nodes and metastasize to other organs, like the brain 2. Due to its prominent hematologic spread, breast cancer screening is highly encouraged in women who exhibit risk factors such as age, family history, and exogenous hormone use 3. Germ-line mutations in high-penetrance breast cancer susceptibility genes like BRCA1, BRCA2, p53 and PTEN have been seen in up to 10% of all breast cancers 4. The pathogenesis of breast cancer includes neoplastic changes of myoepithelial cells that overcome growth suppressor genes and receive nutrient and oxygen supply by angiogenesis and using telomerase to engage in extensive replication of cells 5. A comprehensive electronic search of PubMed/MEDLINE was conducted for studies published from the year 2000 through 2020 utilizing initially broad keywords pertaining to this study including “immunotherapy” and “CAR T-cell” that resulted in over three-thousand articles primarily highlighting the success of CAR T-cell therapy in hematologic diseases. Further narrowing of the search was conducted using additional keywords such as “solid tumor” and “breast cancer,” shortening the results down to 87 articles. This search further showcased the lack of extensive research on the utilization of CAR T-cell therapy in breast cancer and the potential limitations of immunotherapy in solid tumor applications like that of breast and lung.

In 2016, the American Joint Committee on Cancer updated breast cancer staging to include T, N, M, tumor grade, and expression of estrogen and progesterone receptors and HER2 to groups patients into risk categories that help define and guide treatment recommendation 6. For early stage treatment, surgery remains the first treatment recommendation with neoadjuvant chemotherapy 7. The current surgical treatment options include radical mastectomy of the entire breast tissue and axillary lymph nodes and a simple mastectomy including solely the breast removal and axillary procedures used in local control of the disease 8. Unfortunately, many cancers develop resistance to traditional treatment modalities like chemotherapy and radiation. Nevertheless, novel advancements of immunotherapy including antibodies, vaccines, immune checkpoint inhibitors, and CAR T-cell therapy are promising 9. One modality in particular termed CAR T-cell therapy involves the genetic modification of a patient's autologous T-cells to express a CAR specific to a tumor antigen of interest followed by mass proliferation and eventual infusion of these cells performed *ex vivo* 10.

**Figure 1:**
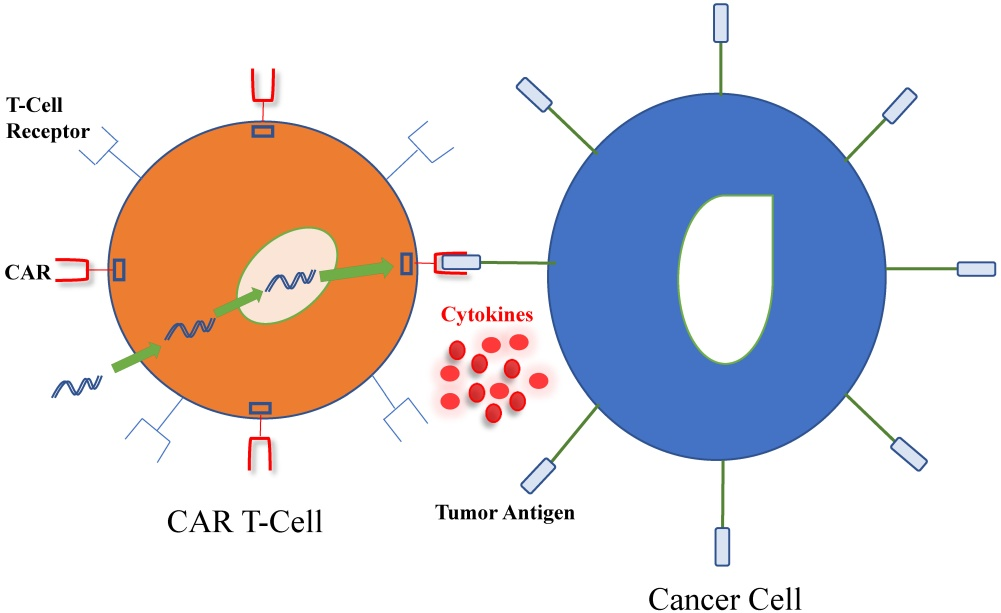
** Interaction between chimeric antigen receptor (CAR) T-cell and cancer cell.** T-cells harvested from patients are genetically modified using viral or non-viral systems to produce the CAR, which includes an antibody-like surface domain, transmembrane domain and intracellular signaling domains. CAR T-cells interact with tumor antigens on cancer cells by using their extracellular surface domain, linked to intracellular costimulatory and signaling domains to amplify the immune response against tumor cells. Furthermore, pro-inflammatory cytokines and chemokines are produced and participate in the eradication of cancer cells.

As described in Figure 1, a tumor-associated antigen (TAA) specific receptor known as CAR is introduced into T-cells, using a plasmid or viral vector, of which lentivirus is the most commonly used 11. CAR combines the specificity of a monoclonal antibody with the inherent cytotoxic and memory capabilities of T-cells. Furthermore, its extracellular domain is derived from the antigen-binding site of a monoclonal antibody and defines CAR's high target affinity while its intracellular domain recapitulates the function of normal T-cells. The incorporation of costimulatory domains, such as CD28 or 4-1BB (CD137) for enhanced survival and proliferation render the CAR T-cells less susceptible than unmodified T cells to negative regulation from tumor cells. Additionally, CAR T-cells do not rely on dendritic cell antigen processing and presentation for its activation. The end effect is that the external targeting domain of CAR binds to the antigen, activating the CAR T- cell and its release of cytokines and other soluble mediators that may directly kill antigen-expressing target cells nearby 12.

Current literature highlights CAR T-cell therapy as a highly effective treatment modality in hematological cancers, like Acute Lymphocytic Leukemia (ALL), exhibiting a 92% full recovery 13. However, in this review, we will explore current strategies and barriers of CAR T-cell therapy in the application to solid tumors, predominately breast cancer.

## Discussion

### Details of CAR-T Therapy

Clinical trials often focus on disseminated tumor cells that lay dormant for years but have metastatic potential in breast cancer patients. Many of these clinical trials involve the triple-negative breast cancer (TNBC) type, a very aggressive malignant type of tumor that is frequently resistant to standard breast cancer therapies due to the absence of estrogen receptors (ER), progesterone receptors, and epidermal growth factor receptors (EGFR), making novel therapeutic modalities such as CAR T-cells particularly attractive 14. To effectively utilize CAR-T against metastatic breast cancer, the identification of an appropriate target antigen and consideration of additional genetic strategies to protect cells from the suppressive tumor microenvironment are critical 15.

As mentioned earlier, modified CAR T-cells contain hybrid receptors that include both an extracellular and intracellular antigen recognizing domain with extensive costimulatory regions that together allow for enhanced antigen affinity 16. The length of the extracellular spacer domain affects tumor cell recognition and CAR T-cell function, likely because of the spatial interaction of CAR and tumor antigen, and the localization of the targeted epitope 17. Additionally, CAR T-cells are major histocompatibility complex (MHC)-independent which excludes human leukocyte antigen (HLA) compatibility issues between donors and recipients. Another particular advantage of CAR T-cells includes their ability to cross the blood-brain barrier, a helpful tool when studying metastases to the central nervous system 18. The activation of resilient T-cells requires three signals: T cell receptor (TCR) (signal 1), co-stimulation (signal 2), and cytokine (signal 3). To enhance activity, persistence, and efficacy, CAR-T therapy has been developed in stages that include first, second, and third generations 19. Originally, first-generation CARs contained only a single signaling domain derived from CD3ζ which may explain the unpromising initial clinical results. Subsequently, second-generation CARs containing an additional co-stimulatory signaling molecule, such as 4-1BB, CD28, CD27, OX40 or ICOS have been developed to stimulate an endogenous immune response against tumor cells via epitope spreading 20. Although second-generation CARs are relatively efficient in the immunosuppressive microenvironment, co-stimulation alone may not be sufficient 21. The latest generations of CAR-T cells, also known as “armored CARs”, contain additional signaling domains from cytokine receptors and express inflammatory cytokines, such as interleukin-12 (IL-12) or IL-18 11.

### Current Targets and Chimeric Antigens

The various types of antigens recognized by CAR T-cells can be extended to carbohydrates and glycolipids that are often modified in cancer cell 22. Additional engineering of CAR T-cells to include intracellular signaling motifs from costimulatory molecules such as CD28, 4-1BB, OX40, and ICOS serve to enhance their proliferation and ability to eradicate cancer cells and cytokine production 23. A folate receptor alpha (FRα) specific chimeric antigen receptor was constructed and the gene encoding this receptor was inserted into T lymphocytes that killed TNBC cells upon amplification and purification 24. One tumor associated antigen called mucin1 (MUC1) is associated with tumor invasiveness and metastatic potential in all cancer cells, including TNBC 25. Immunotherapy was utilized to derive a CAR from the TAB004 monoclonal antibody to bind to an aberrant glycoform of MUC1, called tMUC1. It is important to note that tMUC1 is present in greater than 95% of all TNBC with no significant staining in normal breast epithelium 26. Notably, the T-cells transfected with this specific CAR exhibit antigen-specific cytotoxicity against several TNBC cell lines 14.

Another tumor antigen known as integrin αvβ3 is expressed in several tumor entities including melanoma, glioblastoma, breast, pancreatic, and prostate cancer and promotes tumor cell survival and metastasis 27. Data suggest that adoptive therapy with αvβ3-CAR T-cells has the potential to confer greater therapeutic efficacy compared to immunotherapy with anti-αvβ3 monoclonal antibodies (mABs) through direct cytolytic activity and modification of the tumor microenvironment. The use of immunotherapy in this setting limits tumor angiogenesis by destruction of αvβ3-expressing cancer associated fibroblasts and endothelial cells in tumor-associated blood vessels 28.

One study explored the cell-surface molecule c-Met, which is expressed in ~50% of breast tumors, prompting the construction of a CAR T-cell specific for c-Met in order to halt tumor growth in immune-incompetent mice with tumor xenografts 29. Tumors treated with intertumoral injections of T-cells transduced with c-Met CAR mRNA were excised and analyzed by immunohistochemistry, revealing extensive tumor necrosis at the injection site, cellular debris, loss of c-Met immunoreactivity, all surrounded by macrophages at the leading edges and within necrotic zones. These findings conclude that intertumoral injections of mRNA c-Met-CAR T-cells are well tolerated and elicit an inflammatory response within tumors 30. Similarly, a microphysiologic three-dimensional (3D) lung and breast cancer model that closely resembles the architectural and phenotypical features of primary tumors was used to evaluate the antitumor function of receptor tyrosine kinase-like orphan receptor 1-specific (ROR1-specific) CAR T-cells. Notably, ROR1-CAR T-cells penetrated deep into tumor tissue and eliminated multiple layers of tumor cells located above and below the basement membrane 31. As seen in these studies, much consideration should be made into the use of tumor models to assure safety and efficacy before the clinical application of immunotherapy.

Several CAR T-cell trials are currently enrolling, including trials targeting CEA, HER2, mesothelin, MUC1, NKG2D ligands, ROR1, CD70, and CD133^32^. Among the many tested tumor associated antigens, HER2 is one antigen that is overexpressed in 20% to 30% of all breast cancers, influencing recurrence rates and ultimately survival. Despite the significant improvement in prognosis after therapy with the anti-HER2 antibodies trastuzumab and pertuzumab, a high proportion of patients will eventually experience recurrence even with the synergistic effects of both antibodies when given in the neoadjuvant setting 32,33. Thus, alternative therapies or new combinations to overcome resistance to these antibodies are needed. It was found that dual-targeted T-cells co-expressing a HER2-and MUC-1-specific CAR effectively kill breast cancer cells that normally express both targets. Additionally, engineering T-cells to express a dominant-negative TGFβ receptor that restores T-cell effector function is being investigated in a clinical trial utilizing CAR T-cells that target the HER2 antigen 34.

Another potential target focuses on mesothelin, a cell-surface antigen overexpressed in 67% of TNBC samples 35. Overexpression of mesothelin alone is sufficient to constitutively activate the NFκB, MAPK, and PI3K intracellular pathways promoting tumor cell proliferation and resistance to apoptosis 34. Similarly, the CSPG4 tumor glycoprotein has been identified in 32 of 44 (72.7%) primary TNBC lesions and is inversely correlated with overall survival and time to recurrence. Mechanistically, interactions between chondroitin sulfate side chains of CSPG4 with P-selectin are thought to result in tumor cell activation and augment survival of circulating breast cancer cells 36. CSPG4 presence was also detected in TNBC cancer stem cells, which are regarded as a major source for relapse and resistance 37. Hence, CSPG4-CAR T-cells in TNBC can counteract this mechanism via down-regulating CSPG4 to impair metastasis and stunt breast cancer progression. In aggregate, CSPG4-CAR-T cells have the potential to mount a concerted attack against various targets, including primary TNBC cells, stromal cells, and cancer-associated fibroblasts, which assume a crucial role in maintaining the tumor microenvironment 14.

One study reported on the evaluation of disialoganglioside GD2 expressed in 35.5% of metastatic TNBC as a breast cancer stem cell specific target antigen for immunotherapy 38. Importantly, GD2-CAR-T demonstrated excellent cytolytic activity against GD2 positive cell lines, independent of the tumor entity. This valuable therapeutic option may benefit high-risk breast cancer subtypes like TNBC once clinical trials have proven improved survival rates. Similarly, EGFR-targeted CAR-T cells of the third generation are potent and specific in suppression of TNBC cell growth. This capability was exhibited *in vitro* and *in vivo* in a xenograft mouse model, with minimal off-tumor cytotoxicity. The activation of the interferon γ, granzyme-perforin-PARP and Fas-FADD-caspase signaling pathways in TNBC cells further confirm that CAR-T as an immunotherapy tool may be used to treat TNBC in the clinical setting 39.

TEM8, a marker overexpressed on the vasculature of some solid tumors, has been proposed as a target of controversy 40. A 2018 report stated that T-cells engineered to express a TEM8-specific CAR when injected into mouse models of TNBC, are both safe and effective in controlling tumor growth. This study used the L2 antibody and mouse models of TNBC and reported no toxic effects 41. Conversely, a more recent study by Petrovic presented opposite findings using a panel of TEM8-specific CARs based on the same antibodies, however, causing significant toxicity in healthy mice through the targeting of TEM8 in healthy tissue. Reasons for these contradictory findings could lie in subtle changes in the design of the CAR. For example, different retroviral vectors may have led to different levels of CAR expression per cell 42. This is of particular concern if strategies are employed to enhance the anti-tumor effects of the CAR, for example through dose escalation or increasing the levels of CAR expression and/or function in humans 43.

An intriguing antigen that is overexpressed in most cancers including breast cancer is the human endogenous retrovirus family K (HERV-K) 44. We recently reported that HERV-K is expressed at especially high levels in the basal breast cancer subtype, which is similar to TNBC 45. In contrast to most tumor-specific antigens, HERV-K is absent in nearly all normal human tissues 46. We recently evaluated CAR T-cells targeting the HERV-K envelope protein (K-CAR T cells) for effectiveness in slowing tumor growth in a mouse model of breast cancer 47. CTL assays were employed in *in vitro* studies to determine the cytotoxicity of K-CAR toward MDA-MB-231, a triple-negative breast cancer cell line, and significantly greater lysis was demonstrated for this cell line using K-CAR from breast cancer patients. *In vivo* studies with immunodeficient mice revealed that tumor growth, size, and weights were significantly decreased in mice bearing MDA-MB-231 or MDA-MB-435.eB1 transfectant cancer cell lines, which express a 258-fold increase in the cell growth protein c-erbB-2, were treated with K-CAR T-cells compared to control T-cells or no treatment 48. The mechanism by which K-CAR T-cells repress tumor growth may not be a single mode, since reduced expression of HERV-K in tumor biopsies of the treated mice led to downregulated expression of HERV-K, and this was accompanied by upregulation of p53 and downregulation of its inhibitor MDM2, as well as decreased expression of p-ERK, compared with controls. Anti-HERV-K CAR T-cells have also been investigated for melanoma therapy *in vivo* 49.

### Immunotherapy Combination Therapy

The future of antitumor therapeutics will most likely encompass cell-based therapy in addition to chemotherapy as it is unlikely that CAR T-cell treatment will successfully eradicate a tumor on its own 18. Accurate patient selection and the use of immune checkpoint inhibitors in combination with modalities like photoimmunotherapy that activate the immune system may allow immunotherapy to advance the future of personalized care for breast cancer patients 50. Multiple trials have examined chemotherapy in addition to PD-1/PD-L1 blockade with the goal of enhancing immune priming through antigen release and relieving immunosuppressive signals in the tumor microenvironment. Trastuzumab, a humanized mAb that binds to HER-2 homodimers, is often added to standard chemotherapy to prolong survival and decrease risk of relapse. The addition of Pertuzumab and Trastuzumab to chemotherapy drugs like Taxotere and Carboplatin gives the highest reported clinically partial response (cPR) rate reported to date 51. One clinical trial in Phase 2 has suggested the combination of Pembrolizumab and radiotherapy in patients with metastatic TNBC to be both efficacious and safe for the 17 patients enrolled in the study. However, larger clinical trials of checkpoint blockade plus radiotherapy with predictive biomarkers are required to make a definitive claim 52.

Additionally, two recent reports have linked the gut microbiota composition to the promising clinical response of CAR T-cells in terms of efficacy and toxicity. Specifically, the immunostimulation by intestinal bacteria includes cross-reactions between microbial and tumor antigen stimulation of pattern-recognition receptors (PRRs) and the production of bacterial metabolites that might exert systemic modulatory effects 11. In addition to genetically engineering T-cells to enhance an immune response to tumor infiltration, preconditioning to achieve host lymphodepletion by use of cyclophosphamide, fludarabine, or radiotherapy may promote engraftment of adoptively transferred T-cells. Likewise, CAR T-cell therapy may be used in conjunction with small-molecule inhibitors, monoclonal antibodies, and vaccines 36.

## Challenges

In infant cases of ALL, CAR T-cells achieve up to 90% eradication of the tumor, while in solid tumors, the clinical efficacy is less rewarding due to toxic side effects and fewer clinical trial implementations 19. The mechanism of toxicity includes the immediate release of large quantities of inflammatory cytokines in response to CAR T-cells binding to antigens on target tumor cells. The clinical side effect of such a manifestation is termed cytokine release syndrome (CRS), which is characterized by high fevers, myalgias, nausea, anorexia, and potential hemodynamic or respiratory instability 18. The onset of CRS usually occurs several days after T-cell infusion at the peak of CAR T-cell expansions. The inflammatory cytokines may also activate endothelial cells of the blood-brain barrier, disrupting barrier integrity and driving the CAR-T therapy associated neurotoxicity 53. As IL-6 serves as the primary mediator of CRS, such adverse reactions might respond to IL-6 receptor inhibitors like tocilizumab but this may require further supplementary treatment with corticosteroids to prevent any lethal consequences 54. This so called “on-target/off-tumor effect” of CAR T-cells is mainly caused by T cells further attacking normal cells that express the target antigen causing severe “off-tumor” recognition. Nevertheless, this effect emphasizes the need for identification of tumor specific antigens for proper treatment of solid tumors with CAR T-cells 55. Upon T-cell activation following tumor infiltration, multiple intracellular factors, such as diacylglycerol kinase (DGK), impair T-cell effector functions and promote T-cell anergy. Furthermore, the genetic deletion of DGKζ significantly increases the antitumor activity of mesothelin-targeted CAR T-cells 34.

Another possible concern with cell-based therapies is immunoediting of tumor antigens. As tumor cells are destroyed by CAR T-cells, which are formed from memory cells, they are directed against the tumor antigen they were originally engineered to recognize. In this setting, immunomodulation may prove to be a problem as antigenic shift may cause tumor cells to evolve in a way to create new tumor antigens that may not be recognized by the original CAR T-cells. Thus, this notable antigenic shift, or molecular alteration of an antigen due to recombination, may inadvertently cause CAR T-cells to act against the tumor antigen they were intended to recognize 18. Abnormal vasculature, physical barriers from tumor fibroblasts in the surrounding stroma, and the multitude of immunosuppressive factors such as checkpoint pathways and cytokines prevent efficient solid tumor infiltration of CAR T-cells. The combination of solid tumors secreting chemokines, such as CXCL12 and CXCL5, and the lack of CAR T-cell expression of appropriate chemokine receptors hinder T-cell migration into the tumor site 55. To combat these challenges, novel advancements in trafficking, penetration, and immunosuppressive barriers have been made to improve the efficacy of this mode of therapy. Enhanced expression of chemokine receptors CCR5, CCR2b, CCR4 improve the migration and homing of CAR T-cells to the tumor sites. In terms of penetration, overexpression of heparanase in CAR T-cells may enhance T-cell infiltration by the degradation of heparan sulfate proteoglycans, a key component of the extracellular matrix 56. Likewise, targeting vasculature antigens such as vascular endothelial growth factor receptor (VEGFR-2), integrin alpha V beta 3 (αvβ3) or prostate-specific membrane antigen (PSMA) can also aid CAR T-cell infiltration into the tumor 57. Lastly, secretion of anti-PD-L1 antibodies and IL-12 have shown to improve CAR T-cell function in an immunosuppressive environment. As mentioned earlier, checkpoint inhibitory proteins, such as PD-L1, are upregulated in tumors and the interaction of PD-L1 with its receptor, PD-1 may lead to T-cell exhaustion 19. Under the selection pressure of adoptive cell therapy, cancer cells may evolve to escape the recognition by CD8 + T cells due to epitope mutation and tumor recurrence^1^. For instance, HER2 can undergo proteolysis to cleave the extracellular domain without compromising kinase activity. By using a dual-targeting CAR system, engineered T-cells coexpress two CARs that recognize two distinct antigens so that T-cells can be activated in the presence of either antigen to mitigate antigen-loss escape 20.

Metastasis to the brain from breast cancer poses a significant clinical challenge that may be treated with CAR-based immunotherapy 58. One notable study utilized HER2-CARs containing the 4-1BB costimulatory domain and delivered them intracranially in orthotopic xenograft models to demonstrate robust antitumor efficacy for the treatment of multifocal brain metastases and leptomeningeal disease. This model suggests that regional delivery of CAR T-cells may circumvent systemic targeting of less restricted tumor antigens, like HER2 59.

## Conclusions

While the results of immunotherapy through CAR T-cells have been monumental in cancer therapy, further understanding of the inhibitory tumor microenvironment is needed. Combinatory use of multiple agents in a patient's treatment protocol is found to be advantageous in malignancies like breast cancer that express high immunogenicity and a heterogenous antigen profile. Collectively, studies comparing normal with benign breast tissue indicated early immune infiltration of B cells, T cells, and macrophages as the tumor progresses to ductal carcinoma *in situ* and on to invasive levels. Further exploration as to the direct role of these cells will be imperative in diagnosing breast cancer in its early stages through the identification of discreet cell populations expressing markers commonly associated with a pro-tumor phenotype 60.

The emergence of immuno-oncology has provided a unique opportunity for researchers and clinicians to concentrate on cancers like TNBC that lack targeted therapy universally and are recognized as the most complex and challenging breast cancer subtype to treat, with chemotherapy remaining the standard of care. Therefore, significant effort has been placed on the discovery of novel treatment strategies for subtype TNBC with the hope to broaden this therapeutic approach to other breast cancer subtypes in the future. One strategy in particular targets the use of immune checkpoints for immune escape, specifically the tumor's ability to suppress PD-L1 expression and decrease the activity of cytotoxic T-cells 61. Typically, the binding of PD-L1 to PD-1 on CAR T-cells initiates an inhibitor signal that suppresses the function of CAR T-cells, leading to an exhausted phenotype. Nevertheless, the combination of CAR T-cells with PD-1 inhibitors can overcome tumor microenvironment immunosuppression as demonstrated by Lotfinejad et al. in a murine model of breast cancer. When PD-1 inhibitor antibodies were combined with anti-HER2 CAR T-cells, significant tumor volume reduction was seen and levels of IFN-γ and granzyme B increased, indicating improved immune response 62.

Healthy donor allogeneic CAR T-cells can be derived from an HLA-matched hematopoietic stem cell transplant donor. However, for the non-HLA-matched patient, current efforts to pursue gene-editing approaches using CRISPR/Cas9 have shown to be advantageous. Nevertheless, unwanted side effects of gene editing include off-target cleavage of genes and undesired translocations 63. Ongoing research in the potentially rewarding fields of single-cell genomic analysis 64 and production of next-generation allogeneic CAR T-cells 65 will precisely define the mechanism of action of CAR-T in the breast tumor microenvironment and will provide a ready source of these agents for breast cancer patients. It is also important to consider the fiscal and mental effects of breast cancer. For example, it is expected that patients treated non-operatively are likely to require more intensive imaging follow-up and additional biopsies, potentially leading to increased costs and patient anxiety 66. The potential efficacy of CAR-T therapy is promising and with the speed of technological advances, the hope is that CAR T-cells will be appropriately modified to combat the immunosuppressive forces of the tumor microenvironment and eradicate the solid tumor. The decreasing cost and increasing capacity of sequencing methods could significantly enhance the understanding of complex interactions while engineering the next generation of CAR-T cell therapy for solid malignancies 67.
